# Tandem Exon Duplications Expanding the Alternative Splicing Repertoire

**DOI:** 10.32607/actanaturae.11583

**Published:** 2022

**Authors:** T. M. Ivanov, D. D. Pervouchine

**Affiliations:** Center of Life Sciences, Skolkovo Institute of Science and Technology, Moscow, 121205 Russia

**Keywords:** Alternative splicing, gene structure, tandem exon duplications, RNA-sec

## Abstract

Tandem exon duplications play an important role in the evolution of eukaryotic genes, providing a generic mechanism for adaptive regulation of protein function. In recent studies, tandem exon duplications have been linked to mutually exclusive exon choice, a pattern of alternative splicing in which one and only one exon from a group of tandemly arranged exons is included in the mature transcript. Here, we revisit the problem of identifying tandem exon duplications in eukaryotic genomes using bioinformatic methods and show that tandemly duplicated exons are abundant not only in the coding parts, but also in the untranslated regions. We present a number of remarkable examples of tandem exon duplications, identify unannotated duplicated exons, and provide statistical support for their expression using large panels of RNA-seq experiments.

## INTRODUCTION

The major driving force of molecular evolution is mutation, a process that introduces changes to the genomic sequences that are transmissible through generations. While the most frequent type of mutations are single-nucleotide polymorphisms, which affect single bases, genomic duplications are another important type of DNA changes. A particular subtype are the so-called tandem genomic duplications, which are represented by DNA sequences typically more than 1 kb long, are immediately adjacent to each other, and have a high level of sequence identity [1, 2].

Tandem genomic duplications may affect entire genes, either protein-coding or non-coding, or only gene parts. In the latter case, the duplication leads to propagation of only a portion of the gene sequence, thus affecting the exon-intron structure [3]. The process where the same exon of a gene is duplicated or two or more exons from different genes are brought together ectopically is called exon shuffling [4, 5]. In many cases, exon shuffling through tandem exon duplication has been linked to mutually exclusive exon choice, a regulated pattern of alternative splicing in which only one exon from a group of exons is included in the mature transcript [6, 7]. Mutually exclusive exons (MXEs) are found in the genes across diverse phylae; e.g., cadherin-N (CadN) [8, 9], myosin heavy chain (MHC) [10], 14-3-3*ζ *[ 11], srp [ 12], multidrug resistance-associated protein (MRP) [13] genes in D. melanogaster, mammalian forkhead box (FOX) transcription factor [14] and tropomyosin gene families [15]. Perhaps the most fascinating example of MXE that resulted from tandem duplications is D. melanogaster down syndrome cell adhesion molecule 1 (DSCAM1) gene, which contains 4 groups of MXE clusters, which in total can lead to up to 38,016 distinct protein isoforms [16, 17, 18, 19, 20, 21].

In 2002, a systematic study of common exon duplications and their role in alternative splicing reported that about 10% of animal genes contain tandemly duplicated exons and discovered more than 2,000 unannotated candidate MXEs by similarity searches identifying homology to neighboring exons or within DNA adjacent to exons [22]. However, tandem exon duplications may also affect the intronic and untranslated regions (UTRs) that are not immediately adjacent to annotated exons, and genome annotation databases have significantly expanded. This has motivated us to revisit this question by detecting homology between annotated exons and the entire gene sequences and their genomic neighborhoods. We found that, indeed, tandem exon duplications span far beyond the protein-coding part of gene sequence and are also quite frequent in the untranslated regions. We present a dynamic picture of the abundance of exon duplications as a function of nucleotide sequence homology and report a number of characteristic examples of such duplications.

## METHODS

**Genome sequences and annotations**

The February 2009 (hg19, GRCh37.p13) assembly of the human genome was downloaded from the Genome Reference Consortium [23]. GENCODE comprehensive gene annotation version 19 was downloaded from the GENCODE consortium website [24]. D. melanogaster BDGP Release 6 (dm6) and C. elegans WBcel235 (ce11) genome assemblies were downloaded from the UCSC Genome Browser website [25]. ENSEMBL transcript annotations for D. melanogaster were imported from FlyBase, release dmel_r6.32 [26]. ENSEMBL transcript annotations for C. elegans release 104 were imported from Wormbase [27]. RefSeq transcript annotations for all organisms were downloaded from NCBI RefSeq database [28]. Records other than protein coding genes were excluded from all annotation databases. The numbers of unique exons in the human, D. melanogaster, and C. elegans databases were 329,983; 83,276; and 172,984, respectively.

**Exon homology search**

The homology search was carried out using the EMBL-EBI’s exonerate tool to identify tandem exon duplication [29]. The nucleotide sequence of each exon was aligned to the nucleotide sequence of its parent gene that was extended in both directions by 15% of the gene length in a strand-specific way. We chose to use a percent of the gene length rather than a fixed window around the gene, since human genes are substantially longer than D. melanogaster genes. The choice of 15% cutoff was motivated by the fact that the distance from a gene to its neighbor genes does not exceed 15% of the gene length for approximately one half of D. melanogaster genes. The program was executed in the exhaustive mode to obtain a more accurate alignment. The minimal percent identity cutoff was set to 50%; however, exonerate did not detect sequence homology below 57%. The sequences of the alignments were extracted using the getfasta tool from the bedtools package [30]. The alignments were organized in a bed12 table, in which each line corresponds to one query-target pair (including self-hits). After discarding self-hits, the table contained 116,320; 5,244; and 5,605 query-target pairs for the human, D. melanogaster, and C. elegans genes, respectively.

**Filtering procedure for query-target pairs**

To identify unannotated tandem exon duplications, we filtered the table of query-target pairs using the bedtools intersect utility as follows. We removed the query-target pairs in which the target sequence intersects at least one annotated exon by more than 5% if its length. Additionally, we removed the query-target pairs in which the target sequence intersects at least one annotated interspersed repeat or low-complexity DNA sequence by more than 10% if its length, according to multiple repeats tracks from the UCSC Genome browser [25].

**RNA-seq data**

The RNA-seq data from 6,625 samples in the Genotype-Tissue Expression (GTEx) consortium v7 data were analyzed using the procedure described previously [31]. Short reads were mapped to the human genome using STAR aligner v2.4.2a by the data providers [32]. Split reads supporting splice junctions were extracted using the IPSA package with the default settings [33] (Shannon entropy threshold 1.5 bit). Only split reads with the canonical GT/AG dinucleotides were considered. Uniquely mapped reads were selected based on the presence of NH:1 tag in the BAM files. The average read coverage and PhastCons conservation scores were calculated using the Deeptools software package [34].

## RESULTS

**Nucleotide increase ratio**

**Fig. 1:**
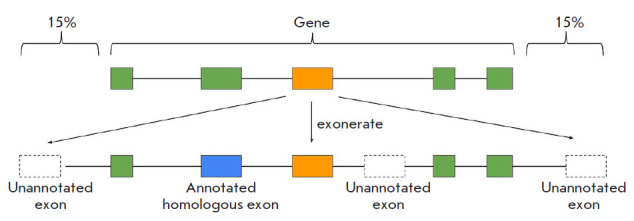
A schematic representation of the tandem exon duplication search. The nucleotide sequence of each exon of every gene is aligned to the nucleotide sequence of its parent gene that is extended 15% in length upstream and downstream

In order to detect exonic duplications, we used the largest to-date exon annotation datasets, including the GENCODE [35] and RefSeq [28] databases, and performed a sequence similarity search for each exon within the extended nucleotide sequence of its parent gene using exonerate software [29]. In what follows, we refer to the annotated exons as query sequences, and their respective homologs that were found by exonerate as target sequences (Fig. 1). Each query-target pair is characterized by the covariates related to the query (e.g., location within CDS or UTR), the covariates related to the target (e.g., whether or not it overlaps an annotated exon), and percent sequence identity between the query and the target. Since many exons are alternatively spliced and, thus, contribute as overlapping regions to the exon annotation sets, we introduced the Nucleotide Increase Ratio (NIR) score, which is defined as the total number of nucleotides covered by the target set as a fraction of the total number of nucleotides covered by the query set in the similarity search with the given or higher percent of sequence identity. By construction, NIR is always greater than 1 since each query serves as its own target with 100% sequence identity. NIR can be computed for all query exons as well as for coding and UTR queries separately. Tables of query-target pairs are available through the online repository https://zenodo.org/record/5474863.

**Fig. 2:**
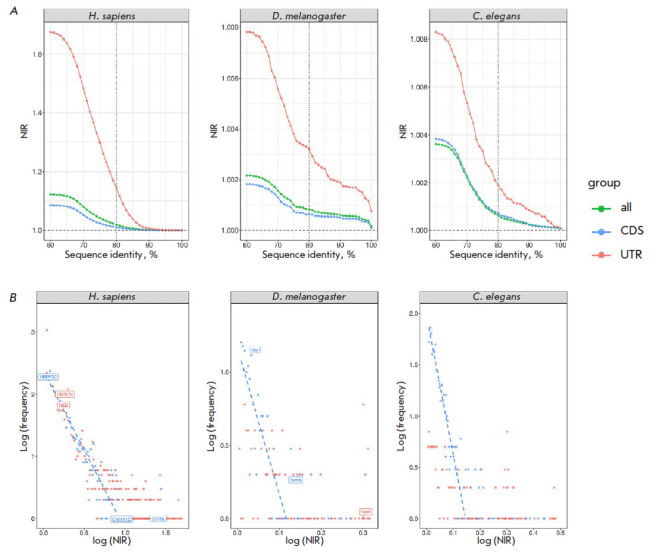
(*A*) The Nucleotide Increase Ratio (NIR) in human, *D. melanogaster*, and *C. elegans *genes as a function of query-target nucleotide sequence identity. (*B*) The frequency distribution of NIR in human, *D. melanogaster*, and *C. elegans *genes (sequence identity threshold 80%). Gene names are shown for remarkable outliers. The insets list the genes with large NIR (cutoffs are shown)

As expected, the NIR values decrease with increasing sequence identity threshold (Fig. 2A). Despite the 50% threshold on minimal sequence identity, exonerate did not detect any query-target pair with sequence homology below 57%. Considering 80% sequence identity cutoff as the midpoint in the 60%–100% interval, which contains all the targets, we observed that approximately 2% of human exonic nucleotides were found to have homologs when performing the similarity search with 80% sequence identity or larger, while only 0.08% of D. melanogaster and 0.06% of C. elegans exonic nucleotides did so. Obviously, this has to do with the fact that the targets of exonic nucleotides beyond the annotated exons belong to intronic regions, and human introns are much longer than those of D. melanogaster and C. elegans. Remarkably, when considering only exons that are located in UTRs, almost 15% of human exonic nucleotides were found to have homologs when performing the similarity search with 80% sequence identity or larger (Fig. 2A). The respective proportions for D. melanogaster and C. elegans were 0.3% and 0.2%, indicating a substantially larger frequency of exon duplications in UTRs.

Next, we asked whether some genes are more prone to tandem exon duplications than the others. To address this question, we computed the NIR values for each annotated gene separately and plotted the NIR frequency distributions (Fig. 2B). The NIR frequencies followed a power law distribution as evidenced by a nearly linear dependence of the logarithm of frequency on the logarithm of the NIR, with a substantial decline towards higher frequencies for larger NIR values in some genes. Interestingly, the human genes with declining NIR values for CDS exons included CAMK1D (Calcium/ Calmodulin Dependent Protein Kinase ID), CLYBL (Citramalyl-CoA Lyase), and NBPF20 (Neuroblastoma breakpoint family member 20) genes; however, some genes also had declining NIR values for the UTRs; e.g., OBSCN (Obscurin, Cytoskeletal Calmodulin and Titin-Interacting RhoGEF) and NEB (Nebulin). In D. melanogaster, the remarkable outliers were the dpy, hydra, and heph genes.

The difference in the propensity of tandem duplications in the genes with large NIR compared to other genes could potentially arise from differences in their exon lengths. To address this, we compared the NIR values in groups of exons equally spaced in ten bins by length. We found that the NIR values decrease approximately fourfold as exon length increases from 20 to 220 nucleotides, thus indicating that longer exons do not contribute to larger NIR values. Indeed, the longer the target, the smaller the likelihood of finding a homolog at 80% sequence identity cutoff should be. Additionally, the average exon length for the top 200 genes with the largest NIR values did not differ significantly from the average exon length in the population of all exons (Wilxcoxon test, P value = 0.2). Therefore, exon lengths do not significantly affect the propensity of tandem duplications. The Gene Ontology analysis of the top 200 genes with large NIR values revealed a statistical enrichment of GO categories related to cell adhesion and nervous system development (biological function), ion binding and receptor activities (molecular function), and membrane localization (cellular compartment).

To further investigate the structure of exonic duplications in these genes, we created a track hub for the UCSC Genome browser as a visualization tool for all query-target pairs. As a positive control, we confirmed that our procedure successfully identified clus ters of tandemly duplicated exons in the genes known from the literature [10, 11, 12, 13] (data not shown). In order to discover novel, unannotated tandem exon duplications, we excluded the query-target pairs that overlap any annotated exon from consideration and filtered out the targets intersecting the annotated repeats or low-complexity regions, since they could have originated through a different mechanism; e.g., exonization of transposed elements [36]. As statistical evidence for the expression of the newfound exons, we computed the read coverage and splice junction support using RNA-seq data from the Genotype Tissue Expression project [31].

## CASE STUDIES

**Obscurin (OBSCN)**

Obscurin (OBSCN) is a remarkable example of a human gene broadly affected by tandem exon duplications. It spans more than 150 kb and contains over 80 exons [37]. The protein encoded by this gene belongs to the family of giant sacromeric signaling proteins, which also includes titin and nebulin [38]. OBSCN is expressed in the heart (RPKM 8.6), prostate (RPKM 2.9), and other tissues [31].

**Fig. 3:**
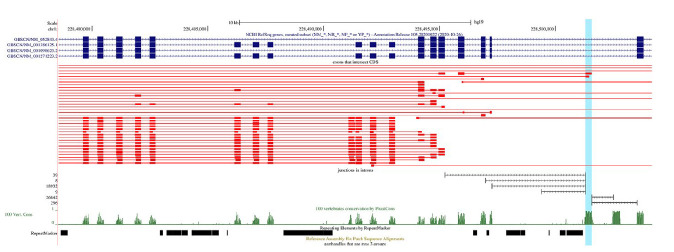
A Genome Browser diagram of tandem exon duplications in *OBSCN*. The annotated transcripts (GENCODE and RefSeq) are shown in dark blue. The query-target pairs with 80% sequence identity are shown in red; query exons are thick, and their targets are thin. The track below query-target pairs represents split reads supporting splice junctions. The PhastCons score over 100 vertebrates is shown in green

Our analysis has shown that the vast majority of OBSCN exons are homologous to each other and similar in length, being indicative of their origin in tandem duplication (Fig. 3). The presence of repeated elements in the intervening introns further suggests that they originated through several rounds of genomic duplications, most likely, via non-homologous recombination. Remarkably, one of the intervening introns contains a region that is homologous to other exons but is not annotated as exon (Fig. 3, blue). The functionality of this region is supported by a peak of phastCons score and the existence of split reads aligning to exon–exon junctions. Interestingly, the same intervening intron contains another peak of phastCons score downstream of the shaded exon that is also supported by split reads; however, it does not show sufficient sequence homology to other exons (percent sequence identity 62.4% vs. 78.9% for the other regions).

**UDP Glucuronosyltransferase Family 1 Member A (UGT1A)**

**Fig. 4:**
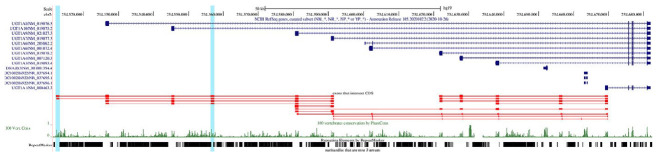
A Genome Browser diagram of tandem exon duplications in *UGT1A*. The color codes in this legend are identical to those in *Fig. 3*

The human UGT1A gene encodes UDP Glucuronosyltransferase Family 1 Member A group of proteins, which is represented by thirteen unique alternate first exons followed by four common exons. UGT1A is associated with diseases including Gilbert syndrome [39] and Crigler–Najjar syndrome [40]. Each first exon encodes the substrate binding site, giving rise to proteins with different N-termini and identical C-termini, and is regulated by its own promoter. According to our analysis, the variable initial exons of these genes are homologous to each other (Fig. 4), thus likely being generated by tandem exon duplications. There is a region in the 5’-UTR of this gene that contains a region that is homologous to the initial exons, but not annotated as an exon. This region is also supported by a peak of phastCons score (Fig. 4, blue). A remarkable feature of this exon cluster is the mutually exclusive choice of the initial exons in the mature transcripts of this gene.

**Examples of tandem exon duplications in D. melanogaster UTRs**

**Fig. 5:**
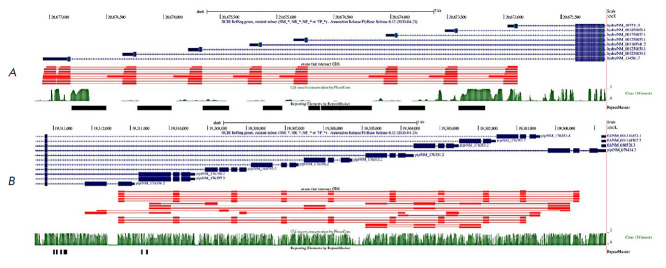
A Genome Browser diagram of tandem exon duplications in *D. melanogaster *genes *hydra *(*A*) and *pip *(*B*) . The color codes in this legend are identical to those in *Fig. 3*

Two remarkable examples of tandem exon duplications in UTRs of D. melanogaster are the hydra (Fig. 5A) and pip (Fig. 5B) genes. In hydra, nine homologous initial exons are spliced in a mutually exclusive manner, while in pip we observe eight tandemly repeated homologous clusters of mutually exclusive terminal exons. It was shown that the initial exon of hydra has undergone recurrent duplications, and seven of these alternative initial exons are flanked on their 3’-side by the transposon DINE-1 (Drosophila interspersed element-1) [41]. At least four of the nine duplicated initial exons can function as alternative transcription start sites [41]. The 3’-UTRs of pip, which encodes sulfotransferase that contributes to the formation and polarity of the embryonic dorsal-ventral axis, have been studied in much less detail. A similar pattern of mutually exclusive usage of 3’- UTRs has been recently reported to be dependent on competing RNA secondary structures, including the 3-UTR of pip [42].

**Expression support by RNA-seq data**

To assess the expression of tandem exon duplications using RNA-seq data, we considered query-target pairs in the human genes in which the target region does not intersect any annotated exon or any annotated repeat element, and merged the remaining targets using the bedtools merge program. This procedure yielded 4,027 intronic targets. Each of these targets was matched randomly to a control region of the same length that was located 30 nt upstream or downstream.

**Fig. 6:**
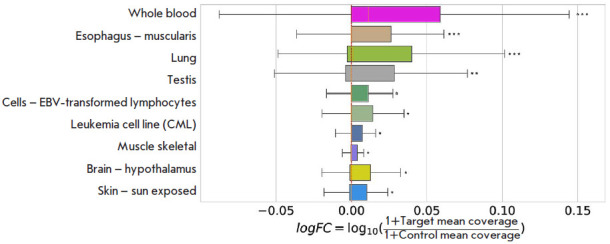
The distribution of the log FCi read coverage metric in GTEx tissues for targets with at least 80% nucleotide sequence homology to the query. The standard color coding of GTEx tissues from [31] was used. Only tissues with significant departure of log FCi from zero are shown (by descending statistical significance). Significance levels were assigned by Wilcoxon signed rank test after the Benjamini-Hochberg correction criteria for multiple testing had been applied

**Fig. 7:**
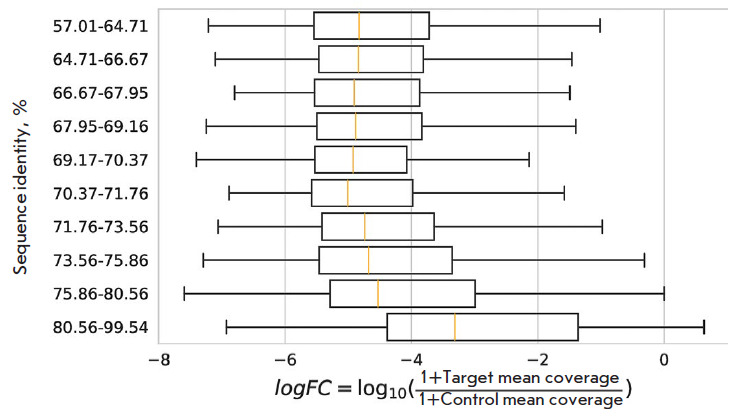
The distribution of the log FCi split read metric. The log FCi metric was computed as log FCi = log10(1 + targeti) – log10(1 + controli), where targeti (controli) is the total number of uniquely mapped split reads supporting the inclusion of target (control) region in tissue i

One inherent problem in assessing the expression of tandem exon duplications using RNA-seq data is that in the case of high nucleotide sequence identity, short reads align equally well to the query and target regions, thus confounding the analysis. We, therefore, filtered out all short reads that aligned to more than one position in the genome and computed the average read coverage for the target and control regions in each of the 53 tissue transcriptomes within the Genotype Tissue Expression (GTEx) project [31] using only uniquely mapped reads. Next, we computed the score log FC_i_ = log_10_(1 + target_i_) - log_10_(1 + control_i_), where target_i_ is the average target read coverage in the tissue i and control_i_ is the average control read coverage in the tissue i. Tissues with an insufficient number of log FC_i_ values (Bladder, Cervix - Endocervix, Cervix - Ectocervix) were excluded from further analysis. In a group of targets that showed at least 80% nucleotide sequence homology to the query, we observed a significant positive departure of the log FCi metric from zero (Wilcoxon signed rank test), which remained significant in some tissues af ter Benjamini-Hochberg correction for multiple testing (Fig. 6); e.g. Whole Blood, Esophagus, Lung, Testis, Muscle, Brain, and also some of the transformed cells. Remarkably, the Wilcoxon signed rank test indicated a statistically significant departure from zero even in the cases when the median was close to zero, which indicates the prevalence of large positive differences in the sample. We also observed an increase in the number of split reads supporting exon–exon junctions for tandemly duplicated exons with higher nucleotide sequence identity (Fig. 7). These results demonstrate that at least some of the unannotated tandemly duplicated exons may indeed be expressed, but in a tissue-specific manner.

Finally, we calculated the difference between average PhastCons [43] scores obtained from the multiple alignments of 100 vertebrate species between the target and control regions. The target regions were on average more evolutionarily conserved than the control regions (Wilcoxon signed rank test, P value = 0.009), which additionally supports their functionality.

## DISCUSSION

An interesting observation made in this work is that tandem exon duplications are prevalent not only in the coding regions, but also in the UTRs of eukaryotic genes and, moreover, they seem to be associated with a mutually exclusive choice of tandemly duplicated initial and terminal exons. A recent study has shown that the regulatory mechanism underlying the mutually exclusive choice of 3’ variable regions in D. melanogaster PGRP-LC pre-mRNA involves competing RNA structures [42]. These RNA structures jointly regulate the 3’ UTR selection through activating the proximal 3’ splice site and concurrently masking the intron-proximal 5’ splice site, together with physical competition of RNA pairing [42]. A similar regulatory program also operates in 3’ variable regions of D. melanogaster CG42235 and pip genes. This observation raises an intriguing question of whether tandem exon duplications in UTRs can generally be controlled by competing RNA structures.

Recently, we have proposed an evolutionary mechanism for the generation of competing RNA structures associated with mutually exclusive splicing via genomic duplications that affect not only exons but also their adjacent introns with stem-loop structures [44]. According to this hypothesis, if one of the two arms of an intronic stem-loop is duplicated, it will automatically generate two sequences that compete for base pairing with another sequence, a pattern that is associated with MXE splicing [13, 14, 15, 21]. This model implies that the mutually exclusive splicing pattern is an inevitable consequence of tandem exon duplications. Considering the high abundance of conserved complementary regions in the UTRs of human genes [45], it appears plausible that tandem exon duplications within UTRs also could generate competing RNA structures leading to mutually exclusive exon inclusion.

## CONCLUSIONS

Tandem exon duplications are abundant not only in the coding parts, but also in the untranslated regions of eukaryotic genes. It still remains an open question whether or not competing RNA structures are broadly involved in the regulation of mutually exclusive splicing of these exons, as well as whether they could be generated as a byproduct of tandem genomic duplications.

## Abbreviations

CDS: coding DNA sequence
UTR: untranslated region
